# Tyrosine Metabolism Pathway Is Downregulated in Dopaminergic Neurons with LRRK2 Overexpression in *Drosophila*

**DOI:** 10.3390/ijms242115587

**Published:** 2023-10-25

**Authors:** Jack Cheng, Bor-Tsang Wu, Hsin-Ping Liu, Wei-Yong Lin

**Affiliations:** 1 Graduate Institute of Integrated Medicine, College of Chinese Medicine, China Medical University, Taichung 40402, Taiwan; t91917@mail.cmuh.org.tw; 2 Department of Medical Research, China Medical University Hospital, Taichung 40447, Taiwan; 3 Department of Senior Citizen Service Management, National Taichung University of Science and Technology, Taichung 40343, Taiwan; wusletter@nutc.edu.tw; 4 Graduate Institute of Acupuncture Science, College of Chinese Medicine, China Medical University, Taichung 40402, Taiwan

**Keywords:** LRRK2, Parkinson’s disease, dopaminergic neuron, tyrosine metabolism

## Abstract

LRRK2 mutations are the leading cause of familial Parkinson’s disease (PD) and are a significant risk factor for idiopathic PD cases. However, the molecular mechanisms underlying the degeneration of dopaminergic (DA) neurons in LRRK2 PD patients remain unclear. To determine the translatomic impact of LRRK2 expression in DA neurons, we employed gene set enrichment analysis (GSEA) to analyze a translating ribosome affinity purification (TRAP) RNA-seq dataset from a DA-neuron-specific-expressing *Drosophila* model. We found that the tyrosine metabolism pathway, including tyrosine hydroxylase (TH), is downregulated in DA neurons with LRRK2 overexpression; in contrast, the Hippo signaling pathway is downregulated in the G2019S mutant compared to wild-type LRRK2 in the DA neurons. These results imply that the downregulation of tyrosine metabolism occurs before pronounced DA neuron loss and that LRRK2 may downregulate the tyrosine metabolism in a DA-neuron-loss-independent way.

## 1. Introduction

Parkinson’s disease (PD), which was first described more than two centuries ago, is a slowly progressing and complex neurological disorder that involves neurodegeneration in the substantia nigra pars compacta (SNpc), leading to dopamine deficiency and typical motor symptoms, along with non-motor symptoms that can precede motor dysfunction [1]. PD is the fastest-growing neurological disease worldwide [2], and its global impact, as measured via mortality rates and disability, has more than doubled over the past two decades [3]. At present, disease-modifying treatments are in pre-clinical or clinical trial phases [4], and PD management focuses on symptomatic treatment that enhances dopamine levels or directly stimulates dopamine receptors [5], since dopamine deficiency might be the only point of convergence among many etiological starting points of complex genetic and environmental cues [2]. If we exclude neuro-regeneration options, which are still underdeveloped and risky, it is clear that disease-modifying treatments must be given in the early stages of the disease and must be customized due to the distinct etiological starting points of PD. Thus, an in-depth understanding of the genetic and environmental cues is essential.

Most PD cases are idiopathic. In contrast, 5–10% of PD cases are familial and associated with mutations in multiple genes, including α-synuclein (*SNCA*), Parkin (*PRKN*), PTEN-induced putative kinase 1 (*PINK1*), DJ-1, VPS35, glucocerebrosidase (*GBA*), and leucine-rich repeat kinase 2 (*LRRK2*) [6]. LRRK2 mutations are the leading cause of familial PD cases and are a significant risk factor for idiopathic PD cases. The LRRK2-G2019S mutation, in particular, accounts for a substantial proportion of familial cases and a smaller percentage of sporadic cases [7]. LRRK2-associated PD closely resembles sporadic PD in terms of age of onset, disease progression, and motor symptoms. Understanding the role of LRRK2 is, thus, crucial for understanding the molecular mechanisms underlying familial and sporadic PD [8].

The *LRRK2* gene encodes a 2527 amino acid, 286 kDa multi-domain protein from the ROCO family [9] that is characterized by a GTPase Ras-like G domain (Roc) and a C-terminal of the Roc domain (COR). Additionally, LRRK2 possesses a serine-threonine kinase domain that can phosphorylate itself [10] and a small group of Rab GTPase substrates [11]. Pathogenic mutations in LRRK2, including the most prevalent, G2019S, are mainly concentrated within the Roc, COR, and kinase domains, resulting in alterations in LRRK2’s biochemical activity [12]. LRRK2 is implicated in various cellular processes, including synaptic vesicle endocytosis, receptor degradation and recycling, anterograde trafficking, and retromer-mediated transmembrane recycling. The regulation of these processes by LRRK2 is closely linked to its phosphorylating target, Rab GTPases [13]. Accordingly, LRRK2-G2019S, the most prevalent kinase-enhancing mutation of LRRK2, results in an aberrant gain of pathological function, including effects on synaptic activity, spine morphology, and persistent forms of synaptic plasticity [14]. However, the molecular mechanisms underlying the degeneration of dopaminergic (DA) neurons in LRRK2-G2019S PD patients remain unclear, and this lack of knowledge is a significant obstacle to understanding the disease’s etiology. The full picture of LRRK2 biology, especially its pathogenic role in PD, is still under intensive investigation.

One strategy to identify the genes and pathways influenced by LRRK2-G2019S is to conduct genome-wide mRNA expression profiling to detect changes in gene expression associated with increased LRRK2 kinase activity. Previous investigations have explored the transcriptional alterations induced by the LRRK2-G2019S mutation in mouse brains [15] and human postmortem brain regions [16]. However, these transcriptome profiles encompassed diverse cell populations present in complex tissues, such as the striatum, cortex, and locus coeruleus, making it challenging to discern specific LRRK2-G2019S-dependent changes in gene expression. Pallos and colleagues focused on the LRRK2-G2019S-dependent gene expression of DA neurons by applying translating ribosome affinity purification (TRAP) combined with RNA-seq [17]. Although they successfully identified several candidate genes that were specifically altered in DA neurons, these candidate genes did not exhibit evident functional or pathway enrichment [17]. Starting from Pallos’ raw data, we took alternative approaches, including different normalization and pathway enrichment methods, to determine the consequences of LRRK2 or LRRK2-G2019S expression in DA neurons.

## 2. Results

**Figure 1:**
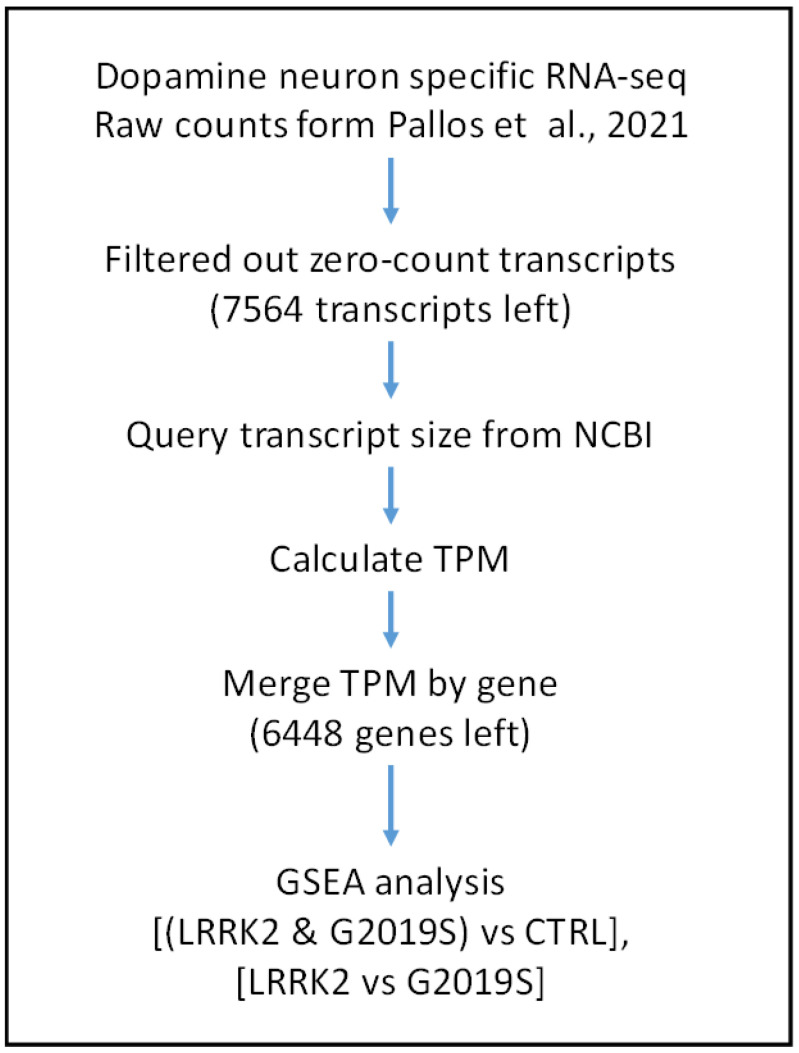
Workflow of this study, with raw data from Pallos et al., 2021 [17].

To elucidate the effects of the DA-neuron-specific expression of LRRK2 or LRRK2-G2019S mutations, we revisited the dataset generated by Pallos and colleagues [17], which utilized the TRAP-seq technique to profile the transcriptome of DA neurons from fruit flies (*Drosophila melanogaster*), with an alternative strategy, as shown in Figure 1. The major differences compared to the original study were the choice of TPM instead of RPKM in counting transcript profiling and the adaptation of gene set enrichment analysis (GSEA). After filtering out the zero-count transcripts in the raw data, we annotated 7564 non-zero transcripts with transcript size (Supplementary Table S1), and TPM was calculated (Supplementary Table S2). Because we intended to use a genetic profile but not a transcript profile in GSEA, we identified a representative genetic TPM if multiple transcripts were available for the gene (Supplementary Table S3). We prepared the genetic TPM in two sets for GSEA, where the *Drosophila* KEGG pathways were used as the gene set. The first set was used to compare LRRK2 (both wild-type and G2019S mutant) to a control (Supplementary Table S4), and the second set was used to compare the G2019S mutant to wild-type LRRK2 (Supplementary Table S5).

**Figure 2:**
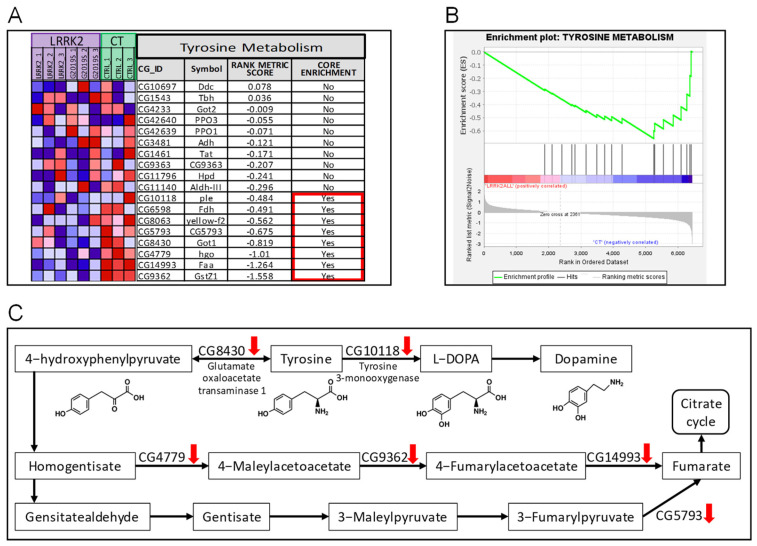
Tyrosine metabolism is downregulated in LRRK2-overexpressing DA neurons. (**A**) Gene expression in the tyrosine metabolism pathway and rank metric scores. Colors of the squares represent the expression level of the gene, with red for high and blue for low expression, respectively. (**B**) Enrichment plot of GSEA analysis showing the characteristic downregulation of the tyrosine metabolism pathway. (**C**) The roles of downregulated genes in the tyrosine metabolism pathway. Red arrows denote downregulation in the LRRK2-overexpressing DA neurons.

For LRRK2 (both wild-type and G2019S mutant) vs. the control, 32 KEGG pathways were upregulated in the LRRK2 samples, where five pathways were significant at FDR < 25% and enriched at nominal *p*-value < 0.05, and three pathways were enriched at nominal *p*-value < 0.01 (Supplementary Table S6), including energy-producing pathways, like starch and sucrose metabolism and valine, leucine, and isoleucine degradation. By contrast, 46 KEGG pathways were downregulated in the LRRK2 samples, where five pathways were significantly enriched at nominal *p*-value < 0.05 (Supplementary Table S7), and only one of them, i.e., the tyrosine metabolism pathway, was enriched at nominal *p*-value < 0.01 and FDR < 25% (Figure 2).

**Figure 3:**
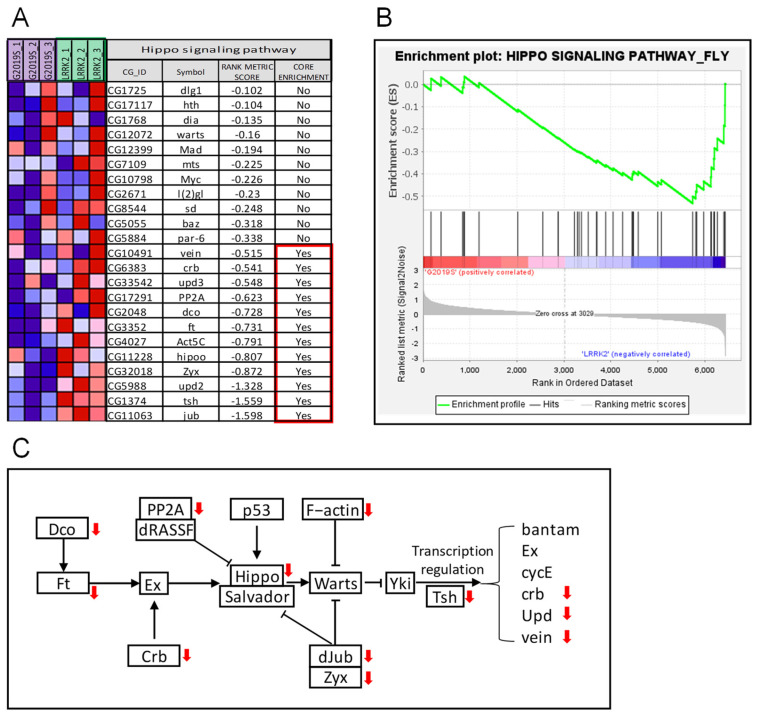
The Hippo signaling pathway is downregulated in G2019S mutant-overexpressing DA neurons compared to its LRRK2-overexpressing counterparts. (**A**) Gene expression in the Hippo signaling pathway and rank metric scores. Colors of the squares represent the expression level of the gene, with red for high and blue for low expression, respectively. (**B**) Enrichment plot of GSEA analysis showing the characteristic downregulation pattern of the Hippo signaling pathway. (**C**) The roles of downregulated genes in the Hippo signaling pathway. Red arrows denote downregulation in the G2019S-overexpressing DA neurons.

For the G2019S mutant vs. LRRK2, 45 KEGG pathways were upregulated in the G2019S samples, where 3 pathways were significantly enriched at nominal *p*-value < 0.05, but none of them were enriched at nominal *p*-value < 0.01 and FDR < 25% (Supplementary Table S8). Meanwhile, 33 KEGG pathways were downregulated in the G2019S samples, where 2 pathways were significantly enriched at nominal *p*-value < 0.05 (Supplementary Table S9), and only 1 of them, i.e., the Hippo signaling pathway, was enriched at nominal *p*-value < 0.01 and FDR < 25% (Figure 3).

**Figure 4:**
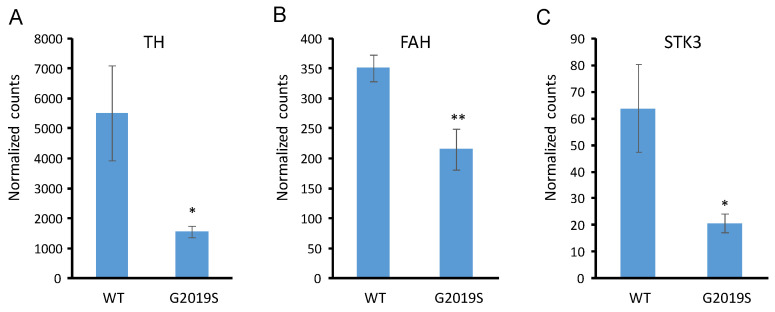
Validation of the dysregulated TH and Hippo in dopaminergic neurons with LRRK2-G2019S. RNA-seq normalized counts of (**A**) TH, (**B**) FAH, and (**C**) STK3 of human iPSC-dopaminergic neurons with wild-type or G2019S LRRK2. TH, tyrosine hydroxylase (homolog of Drosophila CG10118); FAH, fumarylacetoacetate hydrolase (homolog of Drosophila CG14993); STK3, Serine/Threonine Kinase 3 (homolog of Drosophila Hippo). * and ** denote *p*-values < 0.05 and 0.01, respectively. Error bars denote the standard error of the mean. N = 7 and 8 for WT and G2019S groups, respectively.

To validate our findings, we examined an independent dataset of transcript profiling derived from 3D-cultured human iPSC-dopaminergic neurons of healthy subjects and Parkinson’s disease patients (NCBI GEO accession GSE172409) [18]. As shown in Figure 4, the transcription levels of TH and STK3 (the human homolog of Hippo) are significantly decreased in dopaminergic neurons with LRRK2-G2019S.

## 3. Discussion

In this study, we identified dysregulated pathways in LRRK2 transgene DA neurons using gene set enrichment analysis on a TRAP-seq dataset previously published by [17]. Our results revealed that the LRRK2 (both wild-type and G2019S mutant) transgene, compared to control neurons, showed downregulation of the tyrosine metabolism pathway and upregulation of energy-producing pathways. Additionally, G2019S mutant KI, compared to wild-type LRRK2, downregulated the Hippo signaling pathway. The significance of these dysregulations in PD is discussed below.

Tyrosine is an amino acid that is found in various dietary sources and is essential for synthesizing neurotransmitters, like dopamine, norepinephrine, and epinephrine. In the brain, tyrosine is converted into L-DOPA (L-3,4-dihydroxyphenylalanine) by the enzyme tyrosine hydroxylase (*TH*). L-DOPA is further converted into dopamine by the enzyme aromatic L-amino acid decarboxylase (*AADC*) [18]. In Parkinson’s disease, there is a progressive degeneration of DA neurons in the substantia nigra, a brain region responsible for producing dopamine. The loss of DA neurons leads to a significant reduction in dopamine levels in the brain, resulting in the characteristic motor symptoms of Parkinson’s disease, such as tremors, rigidity, bradykinesia, and postural instability [19]. Notably, tyrosine-to-L-DOPA conversion is the rate-limiting step in dopamine synthesis [20]. In this study, we revealed that neurons with transgenic LRRK2 (both wild-type and G2019S mutant) downregulated the transcription level of several enzymes in the tyrosine metabolism pathway (Figure 2), including the bottleneck enzyme, tyrosine 3-monooxygenase (an alternative name for TH). Importantly, as stated by the original contributors of the raw data, the LRRK2 transgene flies were collected at “a time-point where PD-related phenotypes start to manifest but before pronounced DA neuron loss occurs” [17], which may imply that (1) the downregulation of tyrosine metabolism occurs before pronounced DA neuron loss; (2) we may exclude DA neuron loss as the cause of the downregulation of the tyrosine metabolism in this case; (3) we may conclude that the LRRK2 transgene downregulated tyrosine metabolism in a DA-neuron-loss-independent way, at least in this case.

Previous studies have shown that LRRK2 could modulate the homeostasis of DA metabolism. First, LRRK2 is indispensable in DA neurons; LRRK loss-of-function mutants lead to DA neuron degeneration [21]. Secondly, the overexpression of LRRK2 causes Parkinson’s-related phenotypes, which are even more severe when the gain-of-function G2019S mutant is expressed [22]. Thirdly, DA neurons become more numerous early in the life cycle of LRRK2-transgenic *Drosophila* and then fall later, as shown in Figure 2C in a recent work by Zhou and colleagues [23]. These lines of evidence suggest that LRRK2 plays a necessary and delicate role in maintaining the homeostasis of DA metabolism, and our finding fills the knowledge gap concerning the impact of LRRK2 on the tyrosine metabolism pathway before pronounced DA neuron loss during the pathological progress of Parkinson’s disease.

In Parkinson’s disease, patients’ energy expenditure or metabolic rate is often higher than that of healthy individuals [24]. This increased energy expenditure can be attributed to several factors related to the disease and its symptoms, including involuntary muscle movements due to muscle rigidity and tremors, slowness of movement, abnormal gait patterns, and postural instability [25,26]. Higher energy expenditure requires higher energy production. In this study, we found that two pathways related to energy production, i.e., (1) starch and sucrose metabolism and (2) valine leucine and isoleucine degradation, were upregulated in transgenic flies expressing LRRK2 (both wild-type and G2019S mutant) (Supplementary File S6). Although it is unclear whether this upregulation was a direct effect of the LRRK2 transgene or an indirect effect of LRRK2-transgene-induced Parkinson’s-related phenotypes, we propose a dual-hit hypothesis: the LRRK2 transgene has both indirect and direct effects, as a recent study revealed a critical role of LRRK2 in mitochondrial homeostasis [27].

The Hippo signaling pathway is a highly conserved signaling pathway that is crucial in the regulation of cell growth, proliferation, and apoptosis [28]. While its primary functions have been extensively studied in the context of development and cancer, emerging research suggests that the Hippo signaling pathway may also have implications for neurodegenerative diseases. First, the Hippo signaling pathway is involved in regulating cell survival and apoptosis in neurons. Dysregulation of this pathway can lead to an imbalance between cell survival and cell death, contributing to the progressive loss of neurons observed in neurodegenerative diseases [29]. Secondly, activation of the Hippo signaling pathway has been shown to modulate neuroinflammatory responses in the brain [30], and chronic inflammation is a common feature of neurodegenerative diseases. Thirdly, the Hippo signaling pathway and autophagy are reciprocally regulated [31], and autophagy is an active research target in neurodegenerative diseases, especially Parkinson’s disease [32]. In this study, we revealed that transgenic expression of a G2019S mutant, compared to wild-type LRRK2, downregulated the transcription level of several proteins in the Hippo signaling pathway (Figure 3), which may suggest that the G2019S mutant advances PD pathological progress by dysregulating the Hippo signaling pathway.

Currently, no disease-modifying drug is available for PD, and LRRK2-targeting therapy, especially LRRK2 inhibitors, is under extensive research and clinical trials [33]. Our results revealed that the transgenic expression of wild-type or mutant LRRK2 shares common PD-hallmark pathways corresponding to downregulated dopamine synthesis and elevated energy production, which may imply that LRRK2-targeting therapy may also be useful for patients with wild-type but overexpressed LRRK2.

Finally, in the original article [17], 19 differentially expressed genes were identified, including *pav* (*CG1258*), *RfC3* (*CG5313*), *CG6602*, *CG1126*, *CG43799*, *lin-28* (*CG17334*), *CG11068*, *Cc2d2a* (*CG43370*), *Ddx1* (*CG9054*), *l(1)G0196* (*CG14616*), *EfSec* (*CG9841*), *CG2854*, *CR45004*, *MCU* (*CG18769*), *disp* (*CG2019*), *dos* (*CG1044*), *Ugt37c1* (*CG8652*), *Pino* (*CG4710*), and *Cap-D2* (*CG1911*). These 19 genes and our finding of changes to the expression of genes involved in tyrosine metabolism (Figure 2C) and Hippo pathways (Figure 3C) are mutually exclusive. Importantly, our finding was validated by an independent RNA-seq dataset derived from 3D-cultured human iPSC-dopaminergic neurons of healthy subjects and Parkinson’s disease patients (Figure 4), which may suggest that our workflow in this study uncovered the real scientific value of the original data. 

## 4. Materials and Methods

### 4.1. Data Source

The raw counts of DA neuron-specific RNA-seq were downloaded from the original study conducted by Pallos and colleagues [17]. The lengths of transcripts were downloaded from RefSeq, the NCBI Reference Sequence Database [34]. The RNA-seq dataset of 3D-cultured human iPSC-dopaminergic neurons of healthy subjects and Parkinson’s disease patients is available from NCBI GEO with accession number GSE172409 [18].

### 4.2. TPM Calculation

TPM (transcripts per million) was calculated according to [35]. We used the sum of TPM if multiple transcripts were available for one gene. The advantage of using TPM instead of RPKM in cross-sample comparison is briefly described below.

RPKM (Reads Per Kilobase per Million) of a transcript is defined by the number of reads in the transcript, r[t], divided by a thousandth of the length of the transcript, L[t], and a millionth of the total number of total reads in the sample, r[total] [36].

RPKM[t] = (r[t] × 10^9^)/(L[t] × r[total]) (1)

Although RPKM takes the sequencing depth, i.e., the total number of reads of the sample, into consideration, Wagner and colleagues noted that normalization by the total number of reads is not equivalent to the ratios of transcripts, since the total number of transcripts depends on the length distribution of transcripts, which may differ between samples [37], especially those from different phenotypes or treatments. Therefore, Wagner and colleagues proposed TPM, Transcripts Per Million, which is defined by the number of a certain transcript, n[t], divided by a millionth of the total number of all transcripts of the sample, n[total] [37].

TPM[t] = (n[t] × 10^6^)/(n[total]) (2)

The advantage of TPM over RPKM is that TPM eliminates the bias due to the different length distributions of transcripts between samples [37].

### 4.3. Gene Set Enrichment Analysis

The analysis was conducted using GSEA version 4.1.0 [38] with the *Drosophila* KEGG pathway gene set [39]. Two analyses were conducted: (1) (LRRK2 & G2019S) vs. CTRL, and (2) G2019S vs. LRRK2. Common program parameters included permutation type (gene_set), number of permutations (1000), and no collapse to remap gene symbols. Phenotype labels were created at the time of each analysis. For the (LRRK2 & G2019S) vs. CTRL analysis, one group included LRRK2_1, LRRK2_2, LRRK2_3, G2019S_1, G2019S_2, and G2019S_3, and the other group included CT_1, CT_2, and CT3. For the G2019S vs. LRRK2 analysis, one group included G2019S_1, G2019S_2, and G2019S_3, and the other group included LRRK2_1, LRRK2_2, and LRRK2_3.

## 5. Conclusions

In conclusion, the tyrosine metabolism pathway is downregulated in DA neurons with LRRK2 overexpression (both wild-type and G2019S mutant), while the Hippo signaling pathway is downregulated in the G2019S mutant compared to wild-type LRRK2 in the DA-neuron-specific transgene *Drosophila* model.

## Data Availability

All data in this study are included in the Supplementary Materials.

## Supplementary Materials

The following supporting information can be downloaded at: https://www.mdpi.com/article/10.3390/ijms242115587/s1.
